# The European Nucleotide Archive in 2023

**DOI:** 10.1093/nar/gkad1067

**Published:** 2023-11-13

**Authors:** David Yuan, Alisha Ahamed, Josephine Burgin, Carla Cummins, Rajkumar Devraj, Khadim Gueye, Dipayan Gupta, Vikas Gupta, Muhammad Haseeb, Maira Ihsan, Eugene Ivanov, Suran Jayathilaka, Vishnukumar Balavenkataraman Kadhirvelu, Manish Kumar, Ankur Lathi, Rasko Leinonen, Jasmine McKinnon, Lili Meszaros, Colman O’Cathail, Dennis Ouma, Joana Paupério, Stephane Pesant, Nadim Rahman, Gabriele Rinck, Sandeep Selvakumar, Swati Suman, Yanisa Sunthornyotin, Marianna Ventouratou, Senthilnathan Vijayaraja, Zahra Waheed, Peter Woollard, Ahmad Zyoud, Tony Burdett, Guy Cochrane

**Affiliations:** European Molecular Biology Laboratory, European Bioinformatics Institute, Wellcome Genome Campus, Hinxton, Cambridge CB10 1SD, UK; European Molecular Biology Laboratory, European Bioinformatics Institute, Wellcome Genome Campus, Hinxton, Cambridge CB10 1SD, UK; European Molecular Biology Laboratory, European Bioinformatics Institute, Wellcome Genome Campus, Hinxton, Cambridge CB10 1SD, UK; European Molecular Biology Laboratory, European Bioinformatics Institute, Wellcome Genome Campus, Hinxton, Cambridge CB10 1SD, UK; European Molecular Biology Laboratory, European Bioinformatics Institute, Wellcome Genome Campus, Hinxton, Cambridge CB10 1SD, UK; European Molecular Biology Laboratory, European Bioinformatics Institute, Wellcome Genome Campus, Hinxton, Cambridge CB10 1SD, UK; European Molecular Biology Laboratory, European Bioinformatics Institute, Wellcome Genome Campus, Hinxton, Cambridge CB10 1SD, UK; European Molecular Biology Laboratory, European Bioinformatics Institute, Wellcome Genome Campus, Hinxton, Cambridge CB10 1SD, UK; European Molecular Biology Laboratory, European Bioinformatics Institute, Wellcome Genome Campus, Hinxton, Cambridge CB10 1SD, UK; European Molecular Biology Laboratory, European Bioinformatics Institute, Wellcome Genome Campus, Hinxton, Cambridge CB10 1SD, UK; European Molecular Biology Laboratory, European Bioinformatics Institute, Wellcome Genome Campus, Hinxton, Cambridge CB10 1SD, UK; European Molecular Biology Laboratory, European Bioinformatics Institute, Wellcome Genome Campus, Hinxton, Cambridge CB10 1SD, UK; European Molecular Biology Laboratory, European Bioinformatics Institute, Wellcome Genome Campus, Hinxton, Cambridge CB10 1SD, UK; European Molecular Biology Laboratory, European Bioinformatics Institute, Wellcome Genome Campus, Hinxton, Cambridge CB10 1SD, UK; European Molecular Biology Laboratory, European Bioinformatics Institute, Wellcome Genome Campus, Hinxton, Cambridge CB10 1SD, UK; European Molecular Biology Laboratory, European Bioinformatics Institute, Wellcome Genome Campus, Hinxton, Cambridge CB10 1SD, UK; European Molecular Biology Laboratory, European Bioinformatics Institute, Wellcome Genome Campus, Hinxton, Cambridge CB10 1SD, UK; European Molecular Biology Laboratory, European Bioinformatics Institute, Wellcome Genome Campus, Hinxton, Cambridge CB10 1SD, UK; European Molecular Biology Laboratory, European Bioinformatics Institute, Wellcome Genome Campus, Hinxton, Cambridge CB10 1SD, UK; European Molecular Biology Laboratory, European Bioinformatics Institute, Wellcome Genome Campus, Hinxton, Cambridge CB10 1SD, UK; European Molecular Biology Laboratory, European Bioinformatics Institute, Wellcome Genome Campus, Hinxton, Cambridge CB10 1SD, UK; European Molecular Biology Laboratory, European Bioinformatics Institute, Wellcome Genome Campus, Hinxton, Cambridge CB10 1SD, UK; European Molecular Biology Laboratory, European Bioinformatics Institute, Wellcome Genome Campus, Hinxton, Cambridge CB10 1SD, UK; European Molecular Biology Laboratory, European Bioinformatics Institute, Wellcome Genome Campus, Hinxton, Cambridge CB10 1SD, UK; European Molecular Biology Laboratory, European Bioinformatics Institute, Wellcome Genome Campus, Hinxton, Cambridge CB10 1SD, UK; European Molecular Biology Laboratory, European Bioinformatics Institute, Wellcome Genome Campus, Hinxton, Cambridge CB10 1SD, UK; European Molecular Biology Laboratory, European Bioinformatics Institute, Wellcome Genome Campus, Hinxton, Cambridge CB10 1SD, UK; European Molecular Biology Laboratory, European Bioinformatics Institute, Wellcome Genome Campus, Hinxton, Cambridge CB10 1SD, UK; European Molecular Biology Laboratory, European Bioinformatics Institute, Wellcome Genome Campus, Hinxton, Cambridge CB10 1SD, UK; European Molecular Biology Laboratory, European Bioinformatics Institute, Wellcome Genome Campus, Hinxton, Cambridge CB10 1SD, UK; European Molecular Biology Laboratory, European Bioinformatics Institute, Wellcome Genome Campus, Hinxton, Cambridge CB10 1SD, UK; European Molecular Biology Laboratory, European Bioinformatics Institute, Wellcome Genome Campus, Hinxton, Cambridge CB10 1SD, UK; European Molecular Biology Laboratory, European Bioinformatics Institute, Wellcome Genome Campus, Hinxton, Cambridge CB10 1SD, UK; European Molecular Biology Laboratory, European Bioinformatics Institute, Wellcome Genome Campus, Hinxton, Cambridge CB10 1SD, UK

## Abstract

The European Nucleotide Archive (ENA; https://www.ebi.ac.uk/ena) is maintained by the European Molecular Biology Laboratory's European Bioinformatics Institute (EMBL-EBI). The ENA is one of the three members of the International Nucleotide Sequence Database Collaboration (INSDC). It serves the bioinformatics community worldwide via the submission, processing, archiving and dissemination of sequence data. The ENA supports data types ranging from raw reads, through alignments and assemblies to functional annotation. The data is enriched with contextual information relating to samples and experimental configurations. In this article, we describe recent progress and improvements to ENA services. In particular, we focus upon three areas of work in 2023: FAIRness of ENA data, pandemic preparedness and foundational technology. For FAIRness, we have introduced minimal requirements for spatiotemporal annotation, created a metadata-based classification system, incorporated third party metadata curations with archived records, and developed a new rapid visualisation platform, the ENA Notebooks. For foundational enhancements, we have improved the INSDC data exchange and synchronisation pipelines, and invested in site reliability engineering for ENA infrastructure. In order to support genomic surveillance efforts, we have continued to provide ENA services in support of SARS-CoV-2 data mobilisation and have adapted these for broader pathogen surveillance efforts.

## Graphical Abstract

**Graphical Abstract:**
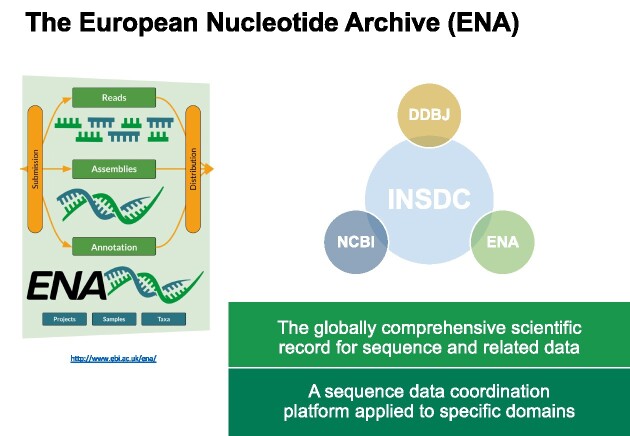

## Introduction

The European Nucleotide Archive (ENA; https://www.ebi.ac.uk/ena) was established in the early 1980s. The ENA provides open access to nucleotide sequences and related information with comprehensive links to other public databases in accordance with the FAIR data principles (1). The ENA offers free access to its data and associated submission, processing, archiving and dissemination services.

The ENA is a founding member of the International Nucleotide Sequence Data Collaboration (INSDC) (2), a long-standing global data exchange initiative. Together with our partners in the NIH-NLM National Center for Biotechnology (NCBI) in the United States (3) and the ROIS DNA DataBank of Japan (DDBJ)(4), we engage with the scientific community as the trusted custodians of freely available public nucleotide sequence data. ENA is also recognised as an ELIXIR Core Data Resource (5), and in 2023 was added to the new collection of Global Core Biodata Resources (https://globalbiodata.org/).

Since its establishment, the ENA has evolved into a globally comprehensive data platform and is used by over 90% of the world's countries. Major areas of work since 2022 (6) include: FAIRness of ENA data, pandemic preparedness and foundational technology. To further enhance the FAIRness of ENA data, we added new minimal requirements for the submission of spatiotemporal annotation of the country of origin and collection date, introduced a tag-based classification system to improve the findability of data with controlled textual annotations, started displaying metadata curations provided by the third parties through the ELIXIR Contextual Data ClearingHouse (https://elixir-europe.org/internal-projects/commissioned-services/establishment-data-clearinghouse), and created the ENA Notebooks platform to quickly call and visualise metadata services by ENA APIs. In anticipation of future pandemics, while ENA infrastructure has continued to support SARS-CoV-2 data mobilisation efforts, it has now been further adapted to support the broader pathogen genomics services being built into the Pathogens Portal (https://www.pathogensportal.org/). In order to scale to meet the ever increasing demands for sequencing data, ENA’s foundational technical enhancements include improvements to the INSDC data exchange and synchronisation pipelines, as well as investing in site reliability engineering approaches. Site reliability engineering is a set of principles and practices that can be applied to software services in order to deliver highly reliable and scalable systems, and ENA is applying these techniques to systematically monitor and improve the performance of ENA services as demand grows exponentially.

In this paper, we outline selected improvements to ENA content and services, outline some of the technical challenges faced by ENA and other sequencing databases as the rate at which life on earth is sequenced grows rapidly, and discuss some of the measures being taken to improve the long-term technical sustainability of ENA.

## Ena content and services

The ENA provides services for the submission, processing, archiving and dissemination of nucleotide sequence data. Data is submitted to the ENA through Webin submission services, and disseminated through the ENA Browser. ENA services are supported by a helpdesk and online user documentation and training materials. The entry points for the key services are listed in Table 1.

**Table 1.** Description of ENA services and their entry points

Service	Description	Entry point
Data submission	Tools and guidelines to submit or to update data with the ENA	https://www.ebi.ac.uk/ena/browser/submit
Data dissemination	Tools and APIs to search, browse, filter and retrieve data from the ENA	https://www.ebi.ac.uk/ena/browser/search
User support	Support form to contact ENA helpdesk for help or feedback	https://www.ebi.ac.uk/ena/browser/support
Documentation	Guidelines and tutorials on how to use the ENA for data submission and retrieval	https://www.ebi.ac.uk/ena/browser/guides

**Figure 1.**
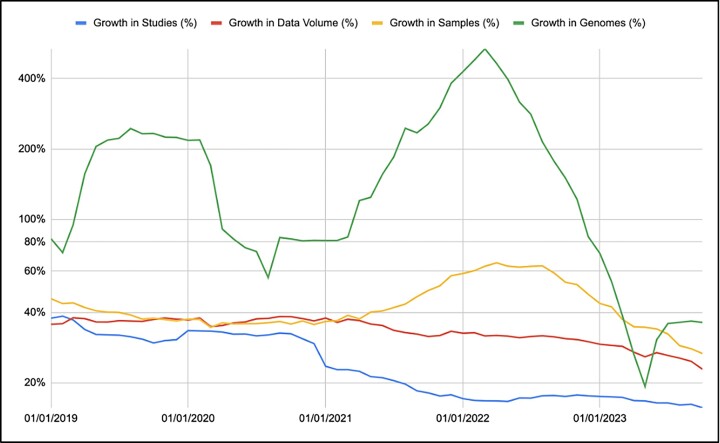
Monthly annualised growth % of studies, samples, genomes and the total data volume between 2019 and 2023 using logarithmic scale. The genome growth peak coincides with a surge in SARS-CoV-2 sequencing.

In the past 12 months, over 2700 submission accounts from 87 countries have deposited data to the ENA, including individual submitters, groups and large-scale brokers. In addition to the submissions exchanged by INSDC partners, 10 thousand studies, over 2.5 million samples, 1 million raw read experiments and over 1.7 million genome assemblies have been captured into ENA since October last year. The total number of raw read experiments in the ENA now exceeds 28 million while the total number of genome assemblies is over 6.5 million. As of October 2023, the total volume of sequence data and annotation in the ENA is more than 50 Petabytes, and has continued to grow at a rate of between 30% and 40% year-on-year for the last 5 years (Figure 1). Projections suggest that the volume of ENA may exceed 150PB in 5 years.

Data is submitted to the ENA using the well established Webin submission services. Webin supports submissions from small-scale to high-throughput, and includes the Webin Portal for interactive submissions (https://www.ebi.ac.uk/ena/submit/webin), Webin RESTful APIs for programmatic submissions (1st version: https://www.ebi.ac.uk/ena/submit/drop-box, 2nd version: https://www.ebi.ac.uk/ena/submit/webin-v2), Webin-CLI for command line submissions (https://github.com/enasequence/webin-cli/releases), and Webin-CLI RESTful APIs for SARS-CoV-2 programmatic submissions (https://www.ebi.ac.uk/ena/submit/webin-cli). For programmatic submitters, Webin also provides reporting (https://www.ebi.ac.uk/ena/submit/report) and authentication (https://www.ebi.ac.uk/ena/submit/webin/auth) services.

Last year, we introduced the second version of the Webin RESTful programmatic submission service (https://www.ebi.ac.uk/ena/submit/webin-v2). We have now extended this service to support submissions of metadata in JSON format. In addition, previously submitted metadata can now be retrieved in the JSON format from the Webin RESTful service. The new JSON format and the existing INSDC XML format are equivalent with a few minor exceptions where deprecated or rarely used XML fields are excluded from the JSON representation. JSON metadata submission instructions and examples are available from: https://ena-docs.readthedocs.io/en/latest/submit/general-guide/programmatic-v2.html.

We have extended the ENA’s CRAM reference registry service. The service was originally created to allow the download of INSDC sequence bases given MD5 checksums to support reference-based sequence compression in the CRAM file format. We have recently extended this service to support the GA4GH Refget API Specification v2.0 (http://samtools.github.io/hts-specs/refget.html) and support both MD5 checksums and INSDC sequence versions. All CRAM reference registry service endpoints are documented here: https://www.ebi.ac.uk/ena/cram/swagger-ui/index.html.

For data dissemination, the ENA Browser is used by >40 000 monthly web visitors, while programmatic consumers make tens of millions of requests to the our RESTful APIs like the ENA Portal API (https://www.ebi.ac.uk/ena/portal/api) and ENA Browser API (https://www.ebi.ac.uk/ena/browser/api) each month. For example, last month ENA Portal API had 34 million requests from 36 000 unique hosts, serving 519GB of metadata, while the ENA Browser API served over 40 million requests from 43K unique hosts, serving 2TB of metadata and data.

For raw reads, we have recently made available generated unmapped BAM files alongside the generated FASTQ files in the ENA Browser and via the API. Together with submitted BAM and CRAM files, this improves access to raw reads in GA4GH-compliant read data formats.

The ENA Advanced Search service underwent a major migration to a more cost-effective and fit-for-purpose backend indexing system. This redesign increases performance and extensibility, and allows new and updated data to become available to consumers significantly faster. The search results also received major improvements including deduplication, better harmonisation, support for new cross-datatype searches (e.g. find all records in a study hierarchy), and improved discoverability of specific types or domains of data via the use of classification tags (refer to https://www.ebi.ac.uk/ena/portal/api/doc for more details).

We have also extended the usability of the ena-file-downloader command line tool (https://github.com/enasequence/ena-ftp-downloader/) by adding direct support for ENA Advanced Search queries. This allows users to directly download all data files associated with records that match a search query of any complexity.

## Selected developments in 2023

### FAIRness of ENA data: spatiotemporal annotation

Since late 2021, the INSDC partners have been committed to increase the richness of metadata associated with nucleotide data records by introducing new minimal requirements: requiring new incoming data submissions to include the source of the sequences in time and space, unless a valid exemption is declared.

The minimal requirement has now been introduced for all incoming biosamples for INSDC data. All samples registered in ENA must now include at least the country and year of their collection or report an exemption. The INSDC missing value reporting controlled vocabulary (https://www.insdc.org/submitting-standards/missing-value-reporting/) has been expanded to allow for ‘reporting level’ terms, and hence we encourage data submitters to utilise these reporting terms to provide more granular reasoning for missing metadata. For comparison, of the samples submitted in the 4 months before implementation began, 70% included country and 52% collection date metadata, whereas in the 4 months following implementation of the new minimal requirement, 92% of the submitted samples included country and 91% collection date (where the remainder reported missing values).

During implementation of the new minimal requirement, we have in addition harmonised reporting of spatiotemporal metadata across all community standards used for ENA sample registration. Going forward, all samples have standardised field names for the country of origin and collection date (Geographic location (country and/or sea); collection date), further improving the FAIRness of ENA data. This change will significantly increase discoverability and usability of INSDC data including all incoming raw datasets, assemblies and sequences linked to source samples. We will continue this commitment into the realm of sequences which are not linked to samples, as INSDC has agreed to introduce the requirement to stand-alone annotated sequence records by the end of December 2024.

### FAIRness of ENA data: tagging system

The idea of ’tagging’ is widely used to label the data with additional properties for more flexible grouping. We have applied this concept to improve the discoverability of data in the ENA. This allows our users to pull focused partitions of the data easily, which would otherwise require potentially long or complicated query.

Tags introduced in ENA Advanced Search are controlled textual annotations on objects, based on metadata information such as sample, taxonomy, or geo-location. The tagging system has proved useful in determining the object membership of certain domain specific data portals. Conversely they can also be used to easily obtain vignettes of data from which to build a new data portal rapidly. For example:

- Users can use ‘marine:high_confidence’ to find all samples that are highly likely to be from the marine environment.
- Users can use ‘xref:worms’ to find all records in ENA data that have a corresponding record in the WoRMS (https://www.marinespecies.org/) database.
- We use ‘pathogen’ to find all pathogenic samples, driving the data coverage in the new Pathogens Portal ourselves.

The tags are typically assigned by automatic processes analysing the metadata curated by the ENA team or supplied by third parties around an object. For example, the identification of ‘marine’ sample records is systematically assessed by a combination of geo-coordinates and taxonomic evidence. We can further qualify such identification by a level of confidence which is dictated by a combination of the evidence available on the record to support said assertion. This is an evolving and continuously improving process, where the algorithms and the rule-sets used for classification can be updated as new insights are obtained and thus results in the assigned tags being regularly refreshed. The flexibility of this system allows for new classifications to be easily created allowing the definition of new, high-level contextual groupings for ENA data making the process of discovery more intuitive for certain user communities.

### FAIRness of ENA data: third-party metadata in the ENA Browser

With the ELIXIR Contextual Data Clearinghouse (https://www.ebi.ac.uk/ena/clearinghouse/api/), users can correct or add missing metadata to published data/metadata records. The ClearingHouse is a service that aims to provide a seamless method to exchange curated contextual data between ELIXIR data resources, other data resources and community curation efforts. The ENA has increased visibility of third-party curations provided through the ClearingHouse in the ENA Browser.

### FAIRness of ENA data: ENA Notebooks

For rapid visualisation/analysis of API-based metadata, we have launched a new tool: ENA Notebooks (https://www.ebi.ac.uk/ena/notebooks/). It is designed to facilitate scientific collaborations through an open-source, virtual lab designed to support biodiversity data workflows, discovery, and visualisations. We will be providing further extensions in the near future.

ENA Notebooks serves as a dynamic tool for helping scientists to discover and co-relate biodiversity data, and for providing insights into the interoperability and reusability of data resource APIs. The browser-based application leverages Jupyter Notebooks to create a versatile platform for developing interactive dashboards and reports. ENA Notebooks are well-suited for representing data residing in diverse geographical locations and data stores.

### Support for pathogen genomics data operations

ENA has continued to provide the sequence foundations for the delivery of the European COVID-19 Data Platform (https://www.covid19dataportal.org/the-european-covid-19-data-platform), offering a targeted SARS-CoV-2 data submission service with rapid-response user support. This continues to support the generation of the world's largest set of raw SARS-CoV-2 sequencing data. ENA currently makes available raw sequence data some 6.6 million SARS-CoV-2 isolates and 8.1 assembled sequences from 8.1 million isolates. Collectively, these have provided a key foundation for open science around SARS-CoV-2(7,8).

**Figure 2.**
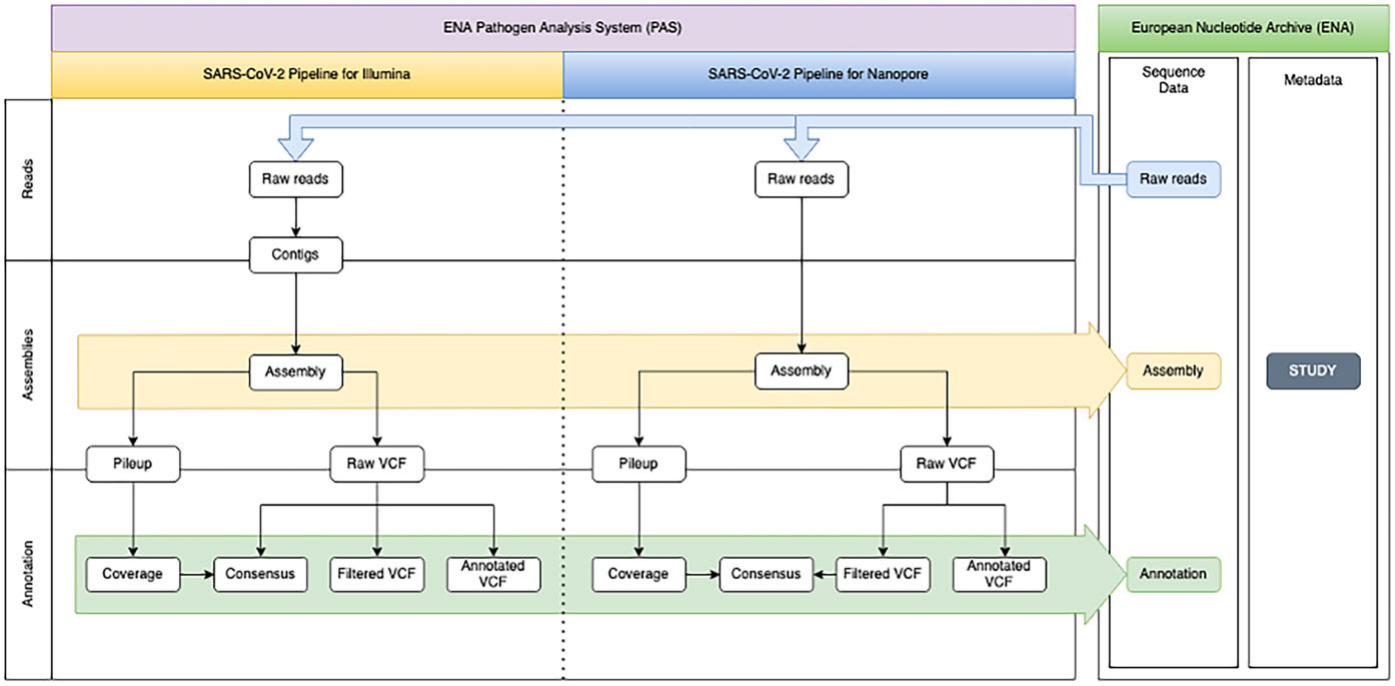
Interaction between SARS-CoV-2 pipelines and ENA is enabled via public APIs only. The ENA public APIs are able to meet the demand for the COVID-19 pandemic. This proves that the ENA public APIs are prepared to support external analysis systems for pandemic at a very large scale with high throughput in the future.

Supporting genomic surveillance, outbreak investigation functions and anti-microbial resistance research, we have further adapted ENA’s services better to support broader pathogens and diseases, including the full integration with the external analysis systems with public APIs (Figure 2). Just as the ENA infrastructure supports the European COVID-19 Data Platform, it now broadens its support to provide the genomics foundation for the recently launched Pathogens Platform (https://www.pathogensportal.org/).

### Foundational Technology: Site reliability engineering

We have adopted site reliability engineering principles and practices to improve the observability and reliability of Webin submission services. We monitor the health of all Webin web services using automated processes in Kubernetes and GCP Monitoring. We use Kubernetes to automatically recover failed services and GCP Monitoring to show service health information on visual dashboards, to create service level objectives (SLOs) and to send automatic alerts. Using GCP Monitoring, we now receive automatic alerts before the availability of any Webin web endpoint falls below our SLO of 99% availability. In addition to Webin submission services, we also collect metrics from Webin processing pipelines.

### Foundational technology: faster INSDC data exchange/synchronisation

As of late of 2022, the majority of the ENA’s data exchange pipelines with INSDC partners were implemented using legacy technology and no longer adequately supported the growing data volume. In the past year we invested significant effort towards improving these processes, particularly aimed at faster synchronisation between partners and the identification and correction of data gaps and discrepancies. Some examples of this work include:

- Using manifest files to inform partners of new/updated records
- Modernising legacy data exchange pipelines and migrating them to the ENA’s own high-performance workflow manager
- Using partner web service APIs to validate data and seek confirmation in ambiguous cases

As a result, the INSDC data exchange has become more efficient, performant, and resilient towards unexpected errors. We can now better monitor the exchange process to detect any anomalies and to take corrective action such as automatic retries when necessary.

### Future work

ENA has established a series of strategic goals for the next several years, and the team will focus its efforts on:

- Improving the reliability and transparency of data submission and presentation services
- Defining scope of the the ENA and reduce complexity of accepted data
- Supporting the integration and use of community recognised sample standards
- Supporting the integration of community recognised standards across non-sample ENA metadata and data types, including experimental designs and protocols
- Expanding the ENA data submissions brokering network
- Collaborating further with our INSDC partners for better sustainability

## Data Availability

ENA services are freely available at (http://www.ebi.ac.uk/ena). Content is distributed under the EMBL-EBI Terms of Use available at (https://www.ebi.ac.uk/about/terms-of-use).
